# Supplementary material for: Predicting visual function by interpreting a neuronal wiring diagram
Source: Nature. 2024 Oct 2;634(8032):113–23. doi: 10.1038/s41586-024-07953-5 (PMC11446822; doi:10.1038/s41586-024-07953-5)
Supplement: Supplementary file 6 — CRF and ERF predictions for individual TmY4 and TmY9 cells. Analogous to Supplementary Data 3, but for TmY target types. Shown are the top four monosynaptic pathways, the strongest pathway passing through each of the top ten intermediary types (ranking from Extended Data Fig. 7), and the trisynaptic pathway Tm1–TmY–Dm3–TmY (see the section entitled Prediction of spatial normalization). [file 41586_2024_7953_MOESM6_ESM.zip › DataS4/TmY9q/720575940631141573.pdf]

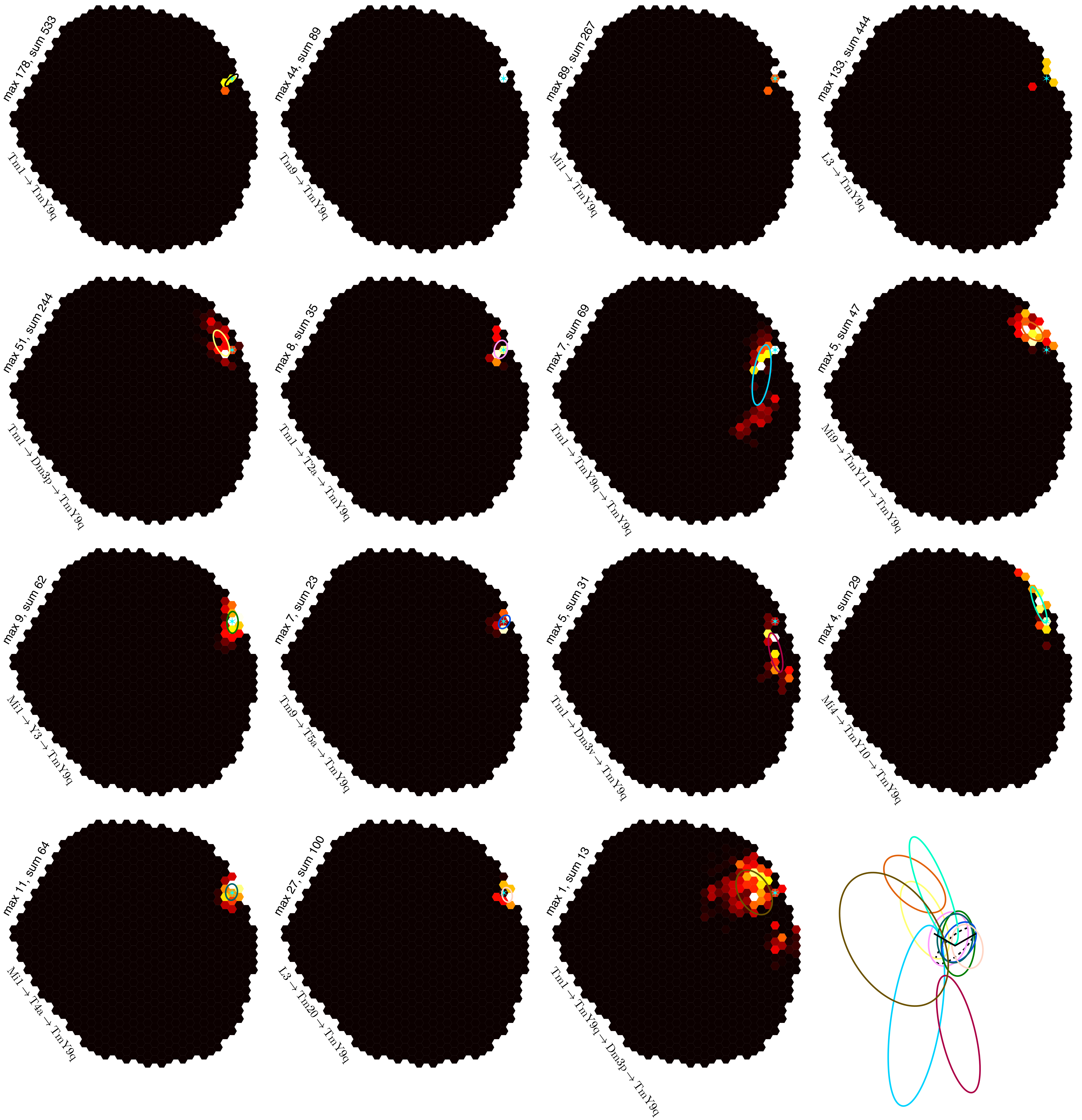

max 178, sum 533
Tm1 → TmY9q
max 44, sum 89
Tm9 → TmY9q
max 89, sum 267
Mi1 → TmY9q
max 133, sum 444
L3 → TmY9q
max 51, sum 244
Tm1 → Dm3p → TmY9q
max 8, sum 35
Tm1 → T2a → TmY9q
max 7, sum 69
Tm1 → TmY9q → TmY9q
max 5, sum 47
Mi9 → TmY11 → TmY9q
max 9, sum 62
Mi1 → Y3 → TmY9q
max 7, sum 23
Tm9 → T5a → TmY9q
max 5, sum 31
Tm1 → Dm3v → TmY9q
max 4, sum 29
Mi4 → TmY10 → TmY9q
max 11, sum 64
Mi1 → T4a → TmY9q
max 27, sum 100
L3 → Tm20 → TmY9q
max 1, sum 13
Tm1 → TmY9q → Dm3p → TmY9q
